# Macrophages and iNOS contribute to the effects of dural prolactin and repeated stress in mouse migraine models

**DOI:** 10.1186/s10194-025-02261-3

**Published:** 2025-12-23

**Authors:** Hao-Ruei Mei, Bianca Mason, Ya-Yu Hu, Aiswarya Saravanan, Shrivatsa Kulkarni, Myan Lam, Shiva Nematgorgani, Joseph B. Lesnak, Michael Burton, Gregory Dussor

**Affiliations:** 1 Department of Neuroscience, The University of Texas at Dallas, Richardson, TX USA; 2 Center for Advanced Pain Studies, University of Texas at Dallas, Richardson, TX USA; 3 Department of Psychology, The University of Texas at Dallas, Richardson, TX USA

**Keywords:** Migraine, Headache, Prolactin, Macrophage, iNOS, Sex differences

## Abstract

**Background:**

Migraine impacts 15% of the global population, predominantly women. Previous studies have shown a role for prolactin in animal migraine models induced by either stimulation of the dura mater or repeated stress exposure. However, the site of prolactin action is not fully known nor are its downstream mechanisms. This study investigated the potential downstream mechanisms and the cell types involved in prolactin- and repeated stress-induced migraine-like responses.

**Methods:**

Two preclinical migraine models were used in this study, dural stimulation and repeated restraint stress. Dural injections in mice enabled drug delivery to the dura mater through the intersection of the lambdoidal and sagittal sutures. Additionally, a model of repeated stress-induced periorbital hypersensitivity and priming to a subthreshold nitric oxide donor was used. Von Frey filaments were used to measure periorbital mechanical thresholds before and after dural administration of prolactin or stress.

**Results:**

Conditional knockout of prolactin receptors in Nav1.8-expressing sensory neurons partially but significantly blocked the periorbital hypersensitivity caused by dural application of prolactin (0.5 µg) to female mice. Depletion of macrophages using clodronate liposome injections before dural prolactin significantly blocked the prolactin responses. The inducible nitric-oxide synthase (iNOS) inhibitor AR-C102222 (ARC; 15 mg/kg) significantly blocked the dural prolactin-induced responses. To determine whether macrophages and iNOS contribute to repetitive stress-induced periorbital hypersensitivity and priming to SNP, clodronate liposomes or ARC were given before or after repetitive stress exposure. Macrophage depletion prior to or following stress significantly inhibited stress-induced periorbital hypersensitivity in both males and females. However, ARC only blocked stress-induced migraine-like behaviors in females.

**Conclusion:**

This study demonstrates that dural prolactin acts through both neuronal and immune cell mechanisms and is dependent on iNOS activity. In response to repeated stress, macrophages contribute to behavioral responses in both sexes while iNOS is only required in females. These findings suggest that interactions between the immune and nervous systems are important for the effects of prolactin and stress on migraine-relevant mechanisms and demonstrate further sex differences in specific pathways.

**Supplementary Information:**

The online version contains supplementary material available at 10.1186/s10194-025-02261-3.

## Introduction

Migraine is a highly prevalent neurological disorder, particularly in women [1]. Across the lifespan, women have longer duration of migraine attacks and experience more significant disability than men [2, 3]. The cause behind the difference in migraine prevalence between males and females is not understood. While prolactin is primarily recognized for its involvement in lactation, accumulating evidence suggests a link between prolactin and migraine [4–10]. Clinical studies show that individuals with migraine have higher serum prolactin levels compared to healthy volunteers, and higher prolactin levels may contribute to greater migraine severity [4, 7]. Preclinical studies have also shown a role for prolactin in animal migraine models induced by stimulation of the dura mater, repeated stress exposure, or repeated sumatriptan administration [7–10]. Despite these findings, a comprehensive understanding of the intricate connection between migraine and prolactin is still lacking.

The trigeminovascular system, which includes afferent trigeminal ganglia (TG) neurons and vessels within the meninges, plays an important role in the headache phase of migraine attacks. The afferent fibers of TG neurons innervate the meninges and its vessels and they project to the central nervous system [11–13]. Prolactin receptors are expressed in both TG sensory neurons and dural immune cells [8, 14, 15]. The location where prolactin is acting to exacerbate migraine is not fully known nor are its downstream mechanisms. Previous studies have shown that prolactin selectively sensitized the TG neurons from female rodents, leading to increased responses induced by capsaicin and increased calcitonin gene-related peptide (CGRP) release induced by mustard oil [8, 16, 17]. These findings suggest prolactin exerts its influence at least in part on neurons within the trigeminovascular system.

Apart from neurons, immune cells, which includes both mast cells and macrophages, are proposed to be involved in the underlying mechanisms of migraine attacks [18]. Mast cells can contribute to local inflammation, vasodilation, and sensitization of adjacent nociceptors through rapid degranulation [18–20]. Macrophages are also proposed to contribute to migraine pathophysiology. They can be recruited and activated during sterile neuroinflammation and generate sustained inflammatory responses that can cause persistent sensitization of pain pathway [18, 21, 22]. Several studies highlight the pivotal role of macrophages, the predominant immune cell population within the mouse dura [23–25], in migraine pathophysiology [18, 26, 27]. These cells may contribute to the effects of prolactin as well as being producers of other migraine-relevant factors such as nitric oxide (NO), a well-known trigger of attacks in people with migraine [28].

In vitro studies have demonstrated that the application of prolactin significantly induced the production of NO and cytokines in macrophages [29–31]. In macrophages, NO is synthesized by inducible nitric oxide synthase (iNOS) [32]. The expression level of iNOS in macrophages also undergoes a substantial increase following a 24-hour incubation with prolactin [29]. We thus hypothesized that the effects of prolactin that we and others have shown in preclinical migraine models may be due in part to the activation of macrophages within the meninges and throughout the body. Prior studies have also shown a connection between stress and prolactin in preclinical migraine models [9, 33], thus we also hypothesized that macrophages may be part of the mechanisms by which stress causes behavioral responses consistent with migraine. Finally, in an attempt to determine how macrophages may be mediating the effects of prolactin and stress, we investigated a potential role of iNOS in these migraine-like behaviors.

## Materials and methods

### Animals

This study used 6- to 8- week-old male and female mice and two strains of mice were used. Wildtype CD-1 mice were purchased from Charles River Laboratories (Wilmington, MA, USA). Mice purchased from Chalres River Laboratories were allowed a minimum of 72 h in the animal facility to acclimate to their new environment after arrival from Charles River Laboratories. Transgenic prolactin receptor conditional knockout (CKO) mice (Nav1.8^cre/−^/Prlr^fl/fl^) in a C57BL6J background and their littermates (Prlr^fl/fl^) were bred in the animal facility at the University of Texas at Dallas [9]. The Prlr^fl/fl^ line was generated by inserting lox sites for deletion of the 4th exon and causing a mRNA-eliminating frame shift as previously described. Genotypes of Prlr^fl/fl^ mice were determined by PCR. The presence of the wild-type *Prlr* allele was detected by primer 1 (5′-TGT CCA GAC TAC AAA ACC AGT GGC-3′) and primer 2 (5′-CAG TGC TCT GGA GAG CTG GC-3′) [34]. Nav1.8-Cre mice on a C57Bl/6 genetic background were maintained in our laboratory and genotyped using the following primers: 5′- GTA GGG GTG ATG GAC AGG AG -3′ (Common), 5′- ATA GAA CCT GGG CAG CTG GT -3′(Wild type Reverse), and 5′- CAG GTT CTT GCG AAC CTC AT -3′(Mutant Reverse). Animals were housed on a 12 h light/dark cycle with food and water *ad libitum*. All animal care and procedures were approved by the Institutional Animal Care and Use Committee at the University of Texas at Dallas.

### Measurement of periorbital mechanical hypersensitivity

Before testing baseline withdrawal thresholds, all animals were placed in paraffin wax-free paper cups in a testing rack for at least 3 days (2 h per day) to familiarize them to the testing environment. Baseline thresholds for periorbital mechanical sensitivity were tested prior to any treatment. Only animals that met baseline (0.6 g) were used in the following experiments. Periorbital mechanical sensitivity was tested by probing the mouse periorbital region with von Frey filaments as previously described [35, 36]. Testing started from 0.07 g and went up until reaching 0.6 g or went down until reaching 0.008 g. A response was defined as a mouse removing/swiping the filament away from its face during application. The 50% response threshold was determined through the Dixon “up-and-down” method [36, 37]. Throughout the experiments described in this manuscript, the researchers were blinded to treatment conditions.

### Repetitive restraint stress paradigm

Repetitive restraint stress protocols were performed as previously described [35]. Animals were restrained in tail vein injection tubes designed for animals 15 to 30 g (Stolting #51338). Mice were placed into the injection tubes with the animal facing the acrylic front and facing the hole in the acrylic front of the tube. Their tail was fit through the adjustable tailpiece. After animals were in the correct position, the movable tailpiece was tightened enough so mice could not move or rotate while allowing enough room for the animal to breathe comfortably. Care was taken to avoid any trauma to the mice due to injuries from moving the disk or from threading the tail through the opening. Mice were checked every 15 to 20 min to ensure that they had not altered their position in the restraint devices and to check on general health. If animals had altered their position, the experimenter readjusted animals by loosening the movable disk without completely removing the animal from the restrainer. Mice were restrained for 2 h per day for 3 consecutive days. The restraint stress started no earlier than 9:30 a.m. and ended prior to 12:00 p.m. Animals above 34 g were not used for stress experiments since they exceed the maximum size accommodated by the restrainer.

### Dural injections

Mouse dural injections were conducted according to previously described methods [36]. Briefly, mice were anesthetized with isoflurane for less than 2 min using a nose cone connected to a vaporizer. The total volume of the dural injection was 5 µl. The injection was performed through modified internal cannula (P1 Technologies, Roanoke, VA, USA, part #8IC313ISPCXC, Internal Cannula, standard, 28 gauge, fit to 0.5 mm). The injector projection length was from 0.5 mm to 0.6 mm measured by digital calipers. This length allowed us to reach dura mater through the intersection of the lambdoidal and sagittal sutures. Care was taken to not cause any damage to the dura mater during injections.

### Flow cytometry

Macrophage populations were assessed 24 h after the second injection of clodronate liposomes or control liposomes. Peritoneal macrophages (PMs) were collected for flow cytometry by injecting 5 mL PM isolation media into the peritoneal cavity, followed by gentle massage of the abdominal walls for a few seconds to dislodge peritoneal cells. The lavage fluid was collected into a 15 mL conical tube on ice. Cells were spun down at 350 g for 5 min at room temperature and the resultant pellet was resuspended in 1x PBS and proceeded to cell staining. First cells stained with a live/dead Zombie Dye (Catalog number:423107, BioLegend, San Diego, CA) for 10 min at room temperature, protected from light. Cells were then spun down at 350 g for 5 min at room temperature and resuspended in flow cytometery staining buffer (TruStain FcX, Biolegend, 422302). To block Fc receptors, cells were incubated with an anti-CD16/CD32 antibody (Catalog number:101302, BioLegend, San Diego, CA) for 10 min at room temperature protected from light. Then cells were incubated with antibodies against CD45 (APC, Catalog number:103111, BioLegend, San Diego, CA) and F4/80 (FITC, Catalog number:123107, BioLegend, San Diego, CA) for 30 min at 4 °C in dark. Cells were then spun down at 350 g for 5 min at room temperature, resuspended in flow cytometry staining buffer, and kept on ice till processing. Flow cytometry data were acquired using a BD LSRFortessa flow cytometer (Franklin Lakes, NJ). To determine changes in macrophage populations, cells were gated for Live (Zombie-), CD45+, and F4/80 + cells.

### Compounds

AR-C 102222, 5-[(4’-Amino-5’,8’-difluorospiro[piperidine-4,2‘(1’*H*)-quinaxolin]-1-yl)carbonyl]-2-pyridinecarbonitrile hydrochloride (Tocris, Bristol, United Kingdom; MedChemExpress, Monmouth Junction, New Jersey, USA) was dissolved in water to generate stock solutions (10 mg/ml). Sodium nitroprusside (SNP; (Sigma Aldrich, St. Louis, MO, USA) was prepared from powder and freshly dissolved in sterile PBS. Liposomal clodronate (Clodrosome^®^) and control liposomes (Encapsome^®^) were purchased from Encapsula NanoSciences LLC (Brentwood, TN, USA). Mice received two intraperitoneal (i.p.) injection of 18.4 mM Clodronate disodium salt (150 µl * 2 Clodrosome^®^), or an equivalent volume of control liposomes (Encapsome^®^) at either a 24-hour or 48- hour interval. Drugs dosed through dural injection were dissolved in synthetic interstitial fluid (SIF; 135mM NaCl, 5mM KCl, 10mM HEPES, 2mM CaCl_2_, 10mM glucose, and 1mM MgCl_2_, pH 7.4, 310 mOsm). Prolactin (INSERM, Paris, France) was diluted in SIF with a total injection volume of 5 µL for dural injection. See Table 1 for information on doses, and administration routes.

**Table 1:** Drugs, doses, and routes of administration

Drug	Dose	Administration route
SNP	0.1 mg/kg	Intraperitoneal
Prolactin	0.5 µg	Dural injection
AR-C102222	3*5 mg/kg	Intraperitoneal
	15 mg/kg	Intraperitoneal
Clodronate Liposome	2*150 µl	Intraperitoneal

### Experimental design and statistical analysis

All data are presented as mean ± standard error of the mean (SEM). All in vivo data were analyzed using two-way analysis of variance (ANOVA) followed by Bonferroni post hoc analysis. Statistical significance was set at *p* < 0.05 and Prism 10 software (GraphPad Software, San Diego, CA, USA) was used to generate graphs and conduct statistical analysis. Flow cytometric data were processed using FlowJo 10.9.0 (BD Biosciences, Franklin Lakes, NJ) and analyized using unpaired t’test. Statistical significance was set at *p* < 0.05 and Prism 10 software (GraphPad Software, San Diego, CA, USA) was used to generate graphs and conduct statistical analysis.

## Results

### Both sensory neurons and macrophages are involved in dural prolactin-induced periorbital hypersensitivity

**Fig. 1:**
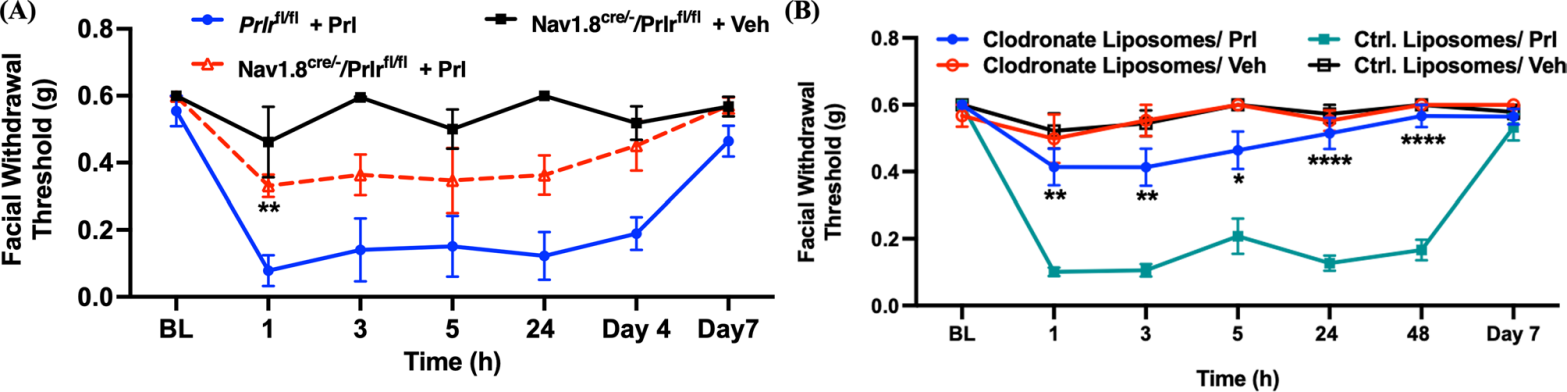
Dural prolactin–induced periorbital hypersensitivity involves contributions from both sensory neurons and macrophages. (**A**) Female prolactin receptor conditional knock-out mice (Nav1.8^cre/−^/Prlr^fl/fl^) or Prlr^fl/fl^ mice received dural administration of 0.5 µg prolactin or vehicle (*n* = 5–6 for each group). Facial withdrawal thresholds were measured before injection and multiple time points after injection. Nav1.8^cre/−^/Prlr^fl/fl^ mice who received dural administration of prolactin showed significantly higher facial withdrawal thresholds compared to Prlr^fl/fl^. who received dural stimulation with prolactin at 1 h after injection. * indicates Prlr^fl/fl^/ Prl vs. Nav1.8^cre/−^/Prlr^fl/fl^/ Prl. Significant differences were determined through Two-way ANOVA followed by Bonferroni multiple comparison analysis. Data are represented as mean ± SEM. **P < 0.01. (**B**) Facial withdrawal thresholds were measured in female mice before and after injection. Female mice received two i.p. injections of 150 µl clodronate liposomes (selective depletion of macrophages) or two i.p. injections of 150 µl of control liposomes 1 and 3 days before dural administered 0.5 µg prolactin or vehicle (*n* = 8–10 for each group). Clodronate liposomes significantly inhibited prolactin-induced facial hypersensitivity at 1, 3, 5, 24, and 48 h post-injection. * indicates Clodronate liposomes/ Prl vs. Ctrl. Liposomes/ Prl. Significant differences were determined through Two-way ANOVA followed by Bonferroni multiple comparison analysis. Data are represented as mean ± SEM. * *P* < 0.05, **P < 0.01, ****P < 0.0001. Ctrl. Liposomes: control liposomes; Prl: prolactin; Veh: vehicle

Previous studies showed that prolactin can act directly on in vitro TG sensory neurons cultured from female mice [8, 38]. However, prolactin receptors are expressed in multiple cell types, such as sensory neurons, immune cells, and endothelial cells [8, 14, 15, 39]. We first aimed to address whether prolactin effects were exclusively mediated by Nav1.8-positive neurons. To investigate this, we evaluated the facial mechanical threshold in prolactin receptor conditional knockout mice (Nav1.8^cre/−^/Prlr^fl/fl^) that received dural administration of prolactin. We used the Cre-loxP mediated conditional knockout strategy, where prolactin receptors were specifically removed from Nav1.8-positive sensory neurons [8, 9, 40–42]. Dural administration of 0.5 µg prolactin only induced responses in female mice but not male mice [8], so only females were used for these experiments. Female Nav1.8^cre/−^/Prlr^fl/fl^ mice or Prlr^fl/fl^ mice received dural administration of 0.5 µg prolactin or vehicle. Nav1.8^cre/−^/Prlr^fl/fl^ mice that received 0.5 µg dural prolactin showed significantly attenuated hypersensitivity 1 h after injection compared to Prlr^fl/fl^ received 0.5 µg prolactin, although there was a non-signficanat effect at 3, 5, 24 h as well as day 4 and 7 after injection compared to Prlr^fl/fl^ (Fig. 1A). These data suggest that responses induced by dural prolactin are not solely mediated by prolactin receptors expressed on Nav1.8-positive neurons but also involve other cell types or remaining prolactin receptors expressed sensory neurons.

TG afferents are able to release neuropeptides which can induce dural immune responses and these afferents also respond directly to proinflammatory molecules released by immune cells. Thus, neuro-immune responses may be involved in the activation of TG afferents and ultimately to migraine pathphysiology [43]. Macrophages are an essential component of the innate immune system. A recent study using single-cell RNA-seq found that macrophages were the most abundant immune cells in mouse dura mater [25]. Prior studies have also shown that exposure of macrophages to prolactin enhanced the production of NO and cytokines [31]. Based on this prior work, we hypothesized that macrophages may be involved in dural prolactin-induced migraine-like responses. To further investigate whether dural prolactin-induced responses involve macrophages, we tested dural injection in mice where macrophages were depleted systemically using administration of clodronate liposomes. Mice received two i.p. injections of clodronate liposomes or control liposome at 24 and 72 h (150 µl each time) before prolactin injection to systemically deplete macrophages. Macrophage depletion through repeated injections of clodronate liposomes was confirmed by flow cytometry (Supplementary Fig. 1).

Following the clodronate liposomes injection protocol, we investigated whether the lack of macrophages attenuated dural prolactin-induced periorbital hypersensitivity. Female mice received dural administration of 0.5 µg prolactin or vehicle 24 h after the second injection of clodronate liposomes or control liposomes. We found that mice given clodronate liposomes before prolactin showed significant block of hypersensitivity (i.e. higher facial withdrawal thresholds) at 1, 3, 5, 24, and 48 h after prolactin injection compared to mice that received control liposomes with prolactin (Fig. 1B). Together, the data from Fig. 1 show that dural prolactin produces periorbital hypersensitivity in part through its receptor expressed on Nav1.8-positive neurons but that its effects are also dependent on the presence of macrophages.

### Dural prolactin-induced periorbital hypersensitivity is blocked by an inhibitor of iNOS

**Fig. 2:**
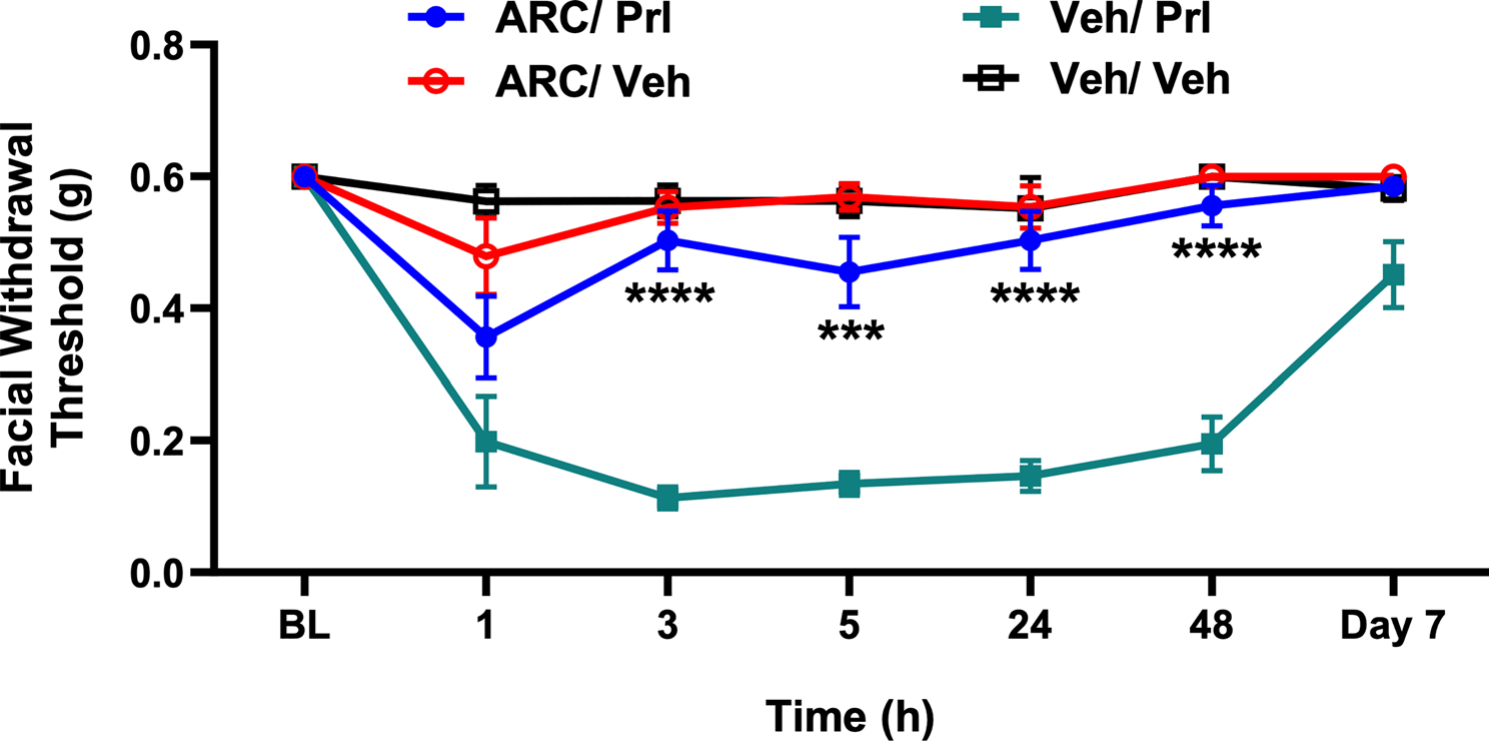
Inhibiting iNOS attenuates dural prolactin-induced periorbital hypersensitivity in female mice. Facial withdrawal thresholds were measured in female mice before and after injection. Mice received i.p. injection of 15 mg/kg AR-C102222 (iNOS inhibitor) or vehicle immediately before dural administration of 0.5 µg prolactin or vehicle (*n* = 8–10 for each group). AR-C102222 significantly inhibited prolactin-induced facial hypersensitivity at 3, 5, 24, and 48 h post-injection. * indicates ARC/ Prl vs. Veh/ Prl. Significant differences were determined through Two-way ANOVA followed by Bonferroni multiple comparison analysis. Data are represented as mean ± SEM. ***P < 0.001, ****P < 0.0001. ARC: AR-C 102,222; Prl: prolactin: Veh: vehicle

Previous studies showed that macrophages incubated with prolactin had significantly higher levels of iNOS expression and NO production [29]. Given that the data shown in Fig. 1B demonstrate a role for macrophages in the effects of dural prolactin, we further investigated whether these responses were mediated through the activation of iNOS. Female mice received i.p. injection of the iNOS inhibitor AR-C102222 (ARC; 15 mg/kg) or vehicle immediately prior to dural injection of prolactin (0.5 µg) or vehicle. Females receiving ARC before prolactin showed significantly reduced hypersensitivity at 3, 5, 24, and 48 h following injection compared to females that received vehicle before prolactin (Fig. 2). These data demonstrate that dural prolactin acts through a mechanism that is dependent on the activity of iNOS.

### Macrophages contribute to repeated stress-induced migraine-like behaviors

**Fig. 3:**
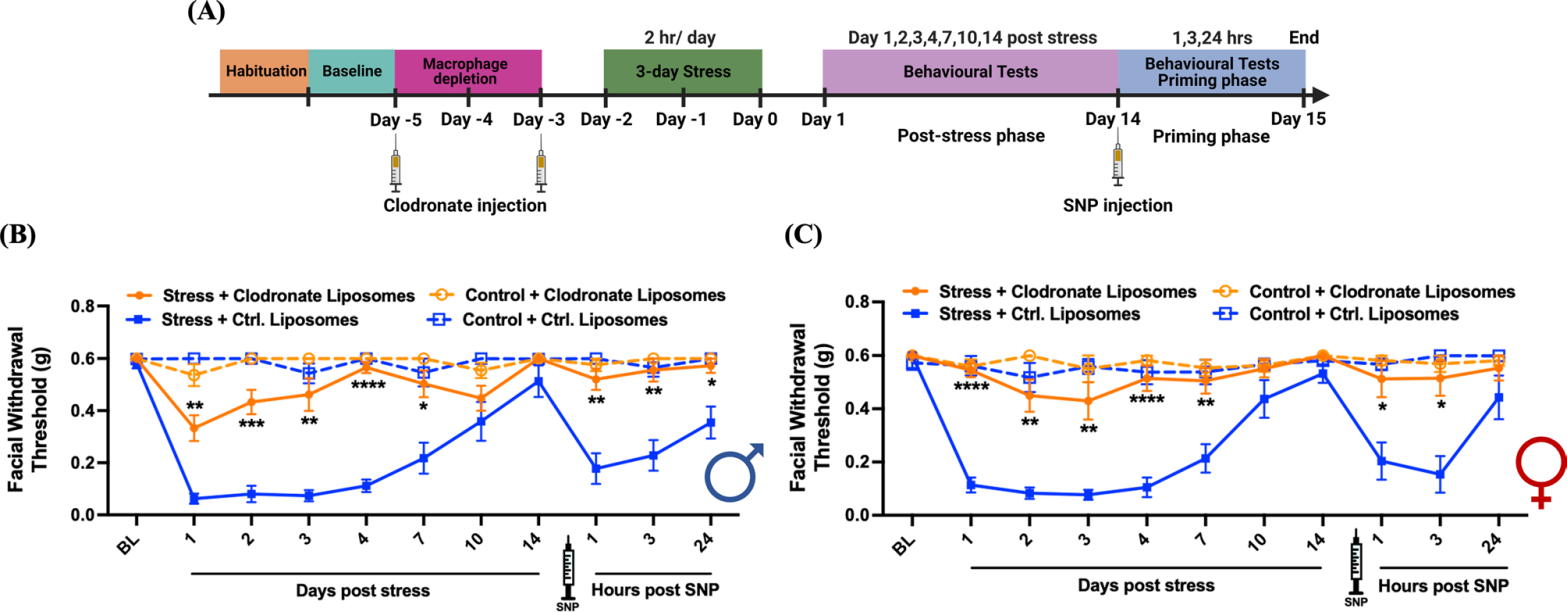
Macrophage depletion prior to stress prevents stress-induced periorbital hypersensitivity. (**A**) The paradigm of clodronate liposomes injections, repeated stress, SNP injection, and testing of facial hypersensitivity in male and female mice. Facial withdrawal thresholds were measured in male and female mice before injection (baseline) and after repeated stress. Mice received two i.p. injections of 150 µl clodronate liposomes (selective depletion of macrophages) or two i.p. injections of 150 µl of control liposomes 1 and 3 days before stress or control procedure. (**B** and **C**) Both male (*n* = 7–9 for each group) and female (*n* = 8–9 for each group) mice who received clodronate liposome injections showed significantly higher facial withdrawal thresholds at 1, 2, 3, 4, and 7 days after stress as well as 1 and 3 h after SNP injection compared to mice received control liposomes injection. * indicates Stress + Clodronate liposomes vs. Stress + Ctrl. Liposomes. Significant differences were determined through Two-way ANOVA followed by Bonferroni multiple comparison analysis. Data are represented as mean ± SEM. *P < 0.05, **P < 0.01, ***P < 0.001, **** *P* < 0.0001. Ctrl. Liposomes: control liposomes; SNP: sodium nitroprusside

We have shown previously that repeated stress primes mice to subthreshold SNP at day 14 post-stress, at time point where facial withdrawal thresholds returned to baseline [35]. Prior work also found that chronic stress led to a significant increase in macrophages in male rat dura compared to naïve rats and this enhancement was maintained at least 24 h after stress [44]. This indicates that stress influences macrophage populations in the dura mater and suggests that macrophages may contribute to stress-related processes. In addition, repeated restraint stress significantly elevated serum prolactin levels in both male and female mice, although to a greater extent in females, and suppression of pituitary prolactin release blocked responses to stress in females but not males [9]. Combined with the role of macrophages in prolactin responses shown in Fig. 1B, we hypothesized that stress responses may also be mediated through the activation of macrophages. To determine whether macrophages contribute to repetitive stress-induced periorbital hypersensitivity and priming to SNP, stressed or control mice received two i.p. injections of clodronate liposomes or control liposomes at 48-hour intervals (150 µl each time) before repetitive stress exposure (Fig. 3A). Both males and females receiving clodronate liposomes before stress showed significant block of hypersensitivity at all time points (1, 2, 3, 4, and 7 days post-stress) after stress compared to mice that received control liposomes before stress. Additionally, clodronate liposome injections before stress significantly blocked the SNP-induced responses in stressed male and female mice at 1, 3, and 24 h post-SNP. No significant differences were found in male and female control (non-stressed) mice post-clodronate liposomes injection or control liposomes injection (Fig. 3B and C). These data demonstrate that following repeated exposure to stress, macrophages contribute to the behavioral responses in both males and females.

**Fig. 4:**
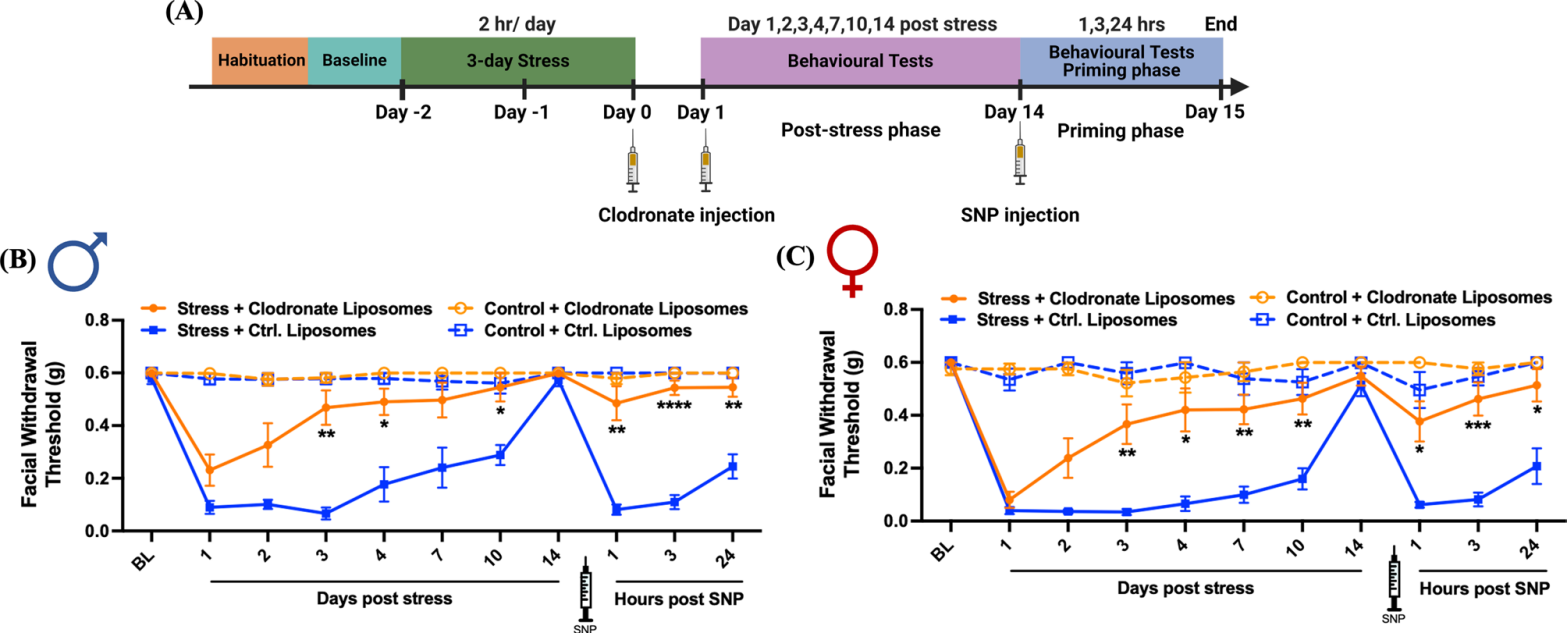
Post-stress macrophage depletion attenuates stress-induced periorbital hypersensitivity. (**A**) The paradigm of clodronate liposomes injections, repeated stress, SNP injection, and testing of facial hypersensitivity in male and female mice. Facial withdrawal thresholds were measured in male and female mice before injection (baseline) and after repeated stress. Mice received two i.p. injections of 150 µl clodronate liposomes (selective depletion of macrophages) or two i.p. injections of 150 µl of control liposomes (Ctrl. Liposomes) after stress on the third day and 24 h after stress. (**B** and **C**) Both male (*n* = 7–8 for each group) and female (*n* = 6–9 for each group) mice that received clodronate liposome injections showed significantly higher facial withdrawal thresholds on 3, 4, 7 (only females), and 10 days after stress as well as 1, 3, and 24 h after SNP injection. * indicates Stress + Clodronate Liposomes vs. Stress + Ctrl. Liposomes. Significant differences were determined through Two-way ANOVA followed by Bonferroni multiple comparison analysis. Data are represented as mean ± SEM. *P < 0.05, **P < 0.01, ***P < 0.001, **** *P* < 0.0001. Ctrl. Liposomes: control liposomes; SNP: sodium nitroprusside

The data described above show that the presence of macrophages at the time of stress exposure is necessary for the full behavioral responses to stress. To determine whether macrophages are still necessary after exposure to stress, we administered stressed or control mice two i.p. injections of clodronate liposomes or control liposomes injection at 24-hour intervals (150 µl each time) after stress exposure was concluded. The shortened injection intervals (24-hours instead of 48-hours) were intended to complete the macrophage depletion process quickly to align with our behavioral test timeline where hypersensitivity behaviors begin to resolve after 3 days in the absence of any interventions. The first timepoint of injection was after stress on the third stress day, and the second timepoint was 24 h following the third stress day (Fig. 4A). In males, injection of clodronate liposomes led to significant block of hypersensitivity at 3, 4, and 10 days after stress. Clodronate liposomes-injected stressed males showed significantly attenuated SNP-induced responses at 1, 3, and 24 h following SNP injection (Fig. 4B). The same effects were also observed in clodronate liposomes-injected females that were stressed. Their behavioral responses at 3, 4, 7, and 10 days after stress as well as 1, 3, and 24 h after SNP injection were significantly reduced compared to control liposome-injected mice (Fig. 4C). No significant differences were detected in male and female control (non-stressed) mice after clodronate liposomes injection or control liposomes (Fig. 4B and C). These data demonstrate that macrophages play a critical role in stress-induced periorbital hypersensitivity in both sexes and that they are important both prior to and following exposure to stress.

### Stress-induced periorbital hypersensitivity requires the activation of iNOS in female but not male mice

**Fig. 5:**
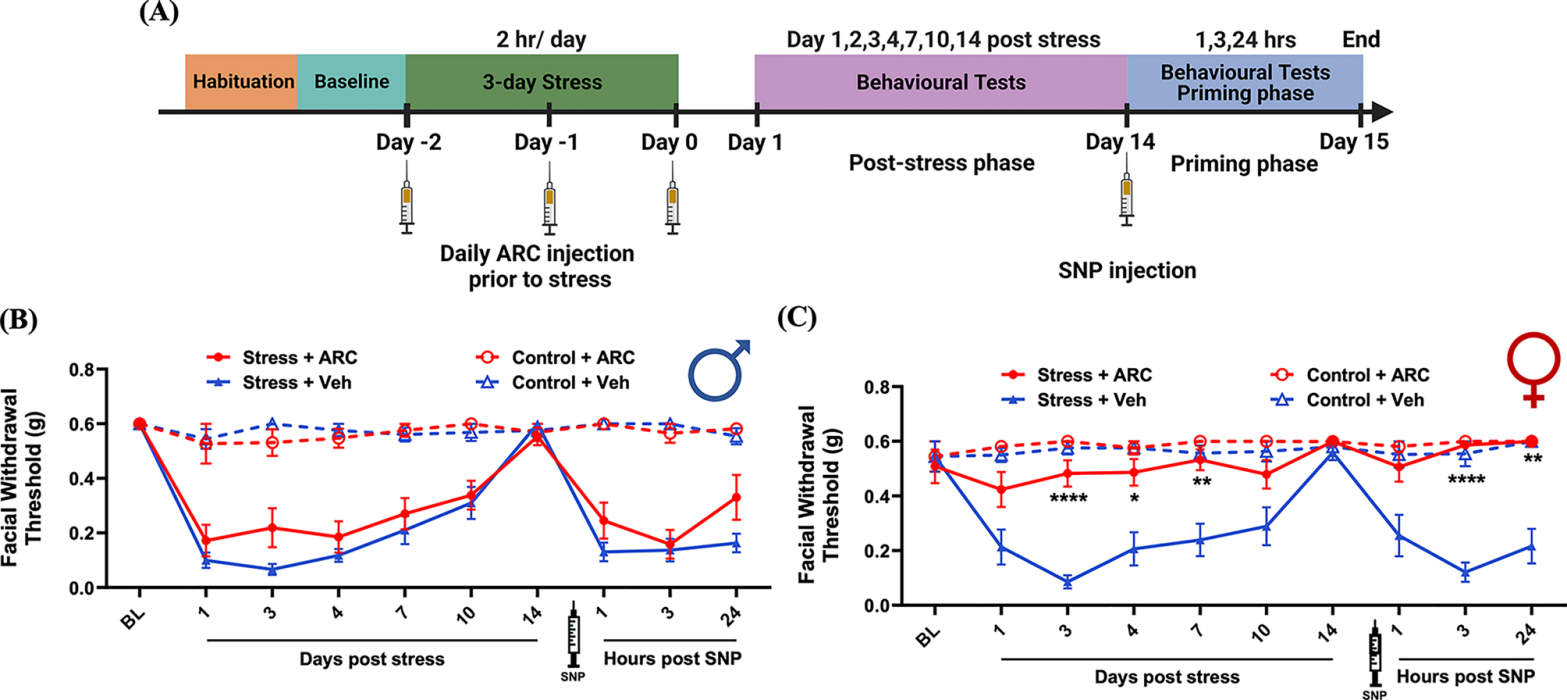
Inhibition of iNOS prior to stress prevents stress-induced periorbital hypersensitivity in female mice. (**A**) The paradigm of ARC injections, repeated stress, SNP injection, and testing of facial hypersensitivity in male and female mice. Facial withdrawal thresholds were measured in male and female mice before injection (baseline) and after repeated stress. Mice received three i.p. injections of 5 mg/kg AR-C102222 (iNOS inhibitor) or vehicle before each stress or control session. (**B**) Stressed male mice who received daily AR-C102222 i.p. injections before repetitive stress showed no significant differences compared to stressed mice who received vehicle injections (*n* = 7–10 for each group). (**C**) Stressed female mice who received daily AR-C102222 i.p. injections before repetitive stress showed significantly higher facial withdrawal thresholds at 3, 4, and 7 days after stress and 3 and 24 h after SNP-injection compared to stressed mice who received vehicle injections (*n* = 9–11 for each group). * indicates Stress/ ARC vs. Stress/ Veh. Significant differences were determined through Two-way ANOVA followed by Bonferroni multiple comparison analysis. Data are represented as mean ± SEM.*P < 0.05, **P < 0.01, **** *P* < 0.0001. ARC: AR-C 102,222; Veh: vehicle

Thus far, this study has shown that the responses to dural prolactin in females are partially mediated by macrophages as well as iNOS activity and that the responses to repeated stress exposure are mediated in part by macrophages in both sexes. Additionally, a prior study using qPCR found that chronic stress enhanced dural iNOS expression in female rats [45] and separately, iNOS was also increased in dural macrophages after nitroglycerin infusion [26]. To investigate whether iNOS contributes to repetitive stress-induced periorbital hypersensitivity and priming to SNP, stressed or control mice received three ip injections of ARC (5 mg/ kg) or vehicle before each of the three stress sessions (Fig. 5A). The ARC dosage was reduced from 15 mg/kg to 5 mg/kg given that we performed repeated injections and thus our aim was to start with a lower dose. In male mice, animals that received 3 total i.p. injections of 5 mg/ kg ARC before stress showed no significant differences at any time points compared to mice that were given vehicle before each stress session (Fig. 5B). In contrast, females that received 3 total i.p. injections of 5 mg/ kg ARC prior to each stress session showed significant block of hypersensitivity at 3, 4, and 7 days after stress compared to mice that received vehicle injection. Females that received 5 mg/ kg ARC before each stress session also showed significantly inhibited SNP-induced periorbital hypersensitivity at 3 and 24 h after SNP injection (Fig. 5C). Both male and female control (non-stressed) mice showed no significant differences after ARC injection compared to mice that received vehicle (Fig. 5B and C).

**Fig. 6:**
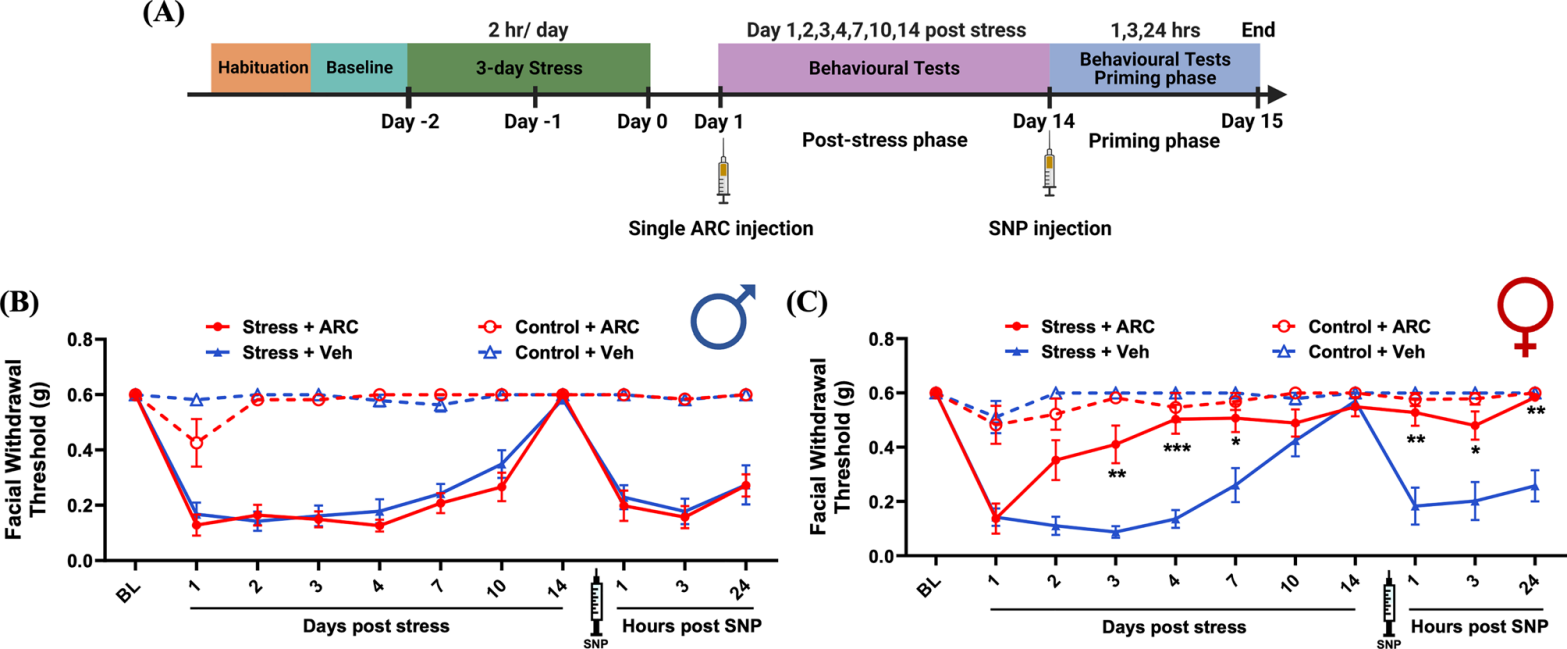
Inhibition of iNOS following stress attenuates stress-induced periorbital hypersensitivity in female mice. (**A**) The paradigm of repeated stress, ARC injections, SNP injection, and testing of facial hypersensitivity in male and female mice. Facial withdrawal thresholds were measured in male and female mice before injection (baseline) and after repeated stress. Mice received single i.p. injections of 15 mg/kg AR-C102222 (iNOS inhibitor) or vehicle 24 h after third stress. (**B**) Stressed male mice who received AR-C 102,222 i.p. injections showed no significant difference compared to stressed mice who received vehicle injections (*n* = 8–10 for each group). (**C**) Stressed female mice who received AR-C 102,222 i.p. injections showed significantly higher facial withdrawal thresholds at 3, 4, and 7 days after stress and 3 and 24 h after SNP-injection compared to stressed mice who received vehicle injections (*n* = 7–10 for each group). * indicates Stress/ ARC vs. Stress/ Veh. Significant differences were determined through Two-way ANOVA followed by Bonferroni multiple comparison analysis. Data are represented as mean ± SEM. *P < 0.05, **P < 0.01, ***P < 0.001. ARC: AR-C 102,222; Veh: vehicle

To further investigate whether iNOS activity is necessary after stress exposure, stress or control animals received a single i.p. injection of 15 mg/kg ARC 24 h after the third-day of stress (Fig. 6A). The male mice that received ARC showed no difference from mice that received vehicle at any time point after stress and after SNP-injection (Fig. 6B). However, female animals that received ARC injection showed significant block of hyperensitivity at 3, 4 and 7 days after stress as well as 1, 3, and 24 h after SNP injection (Fig. 6C). There was no significant difference observed between male and female control (non-stressed) mice following ARC injection when compared to mice that received vehicle (Fig. 6B and C). These findings demonstrate that unlike macrophages whose presence is necessary for behavioral responses to stress in both sexes, iNOS activity is only necessary for stress-induced periorbital hypersensitivity in females.

## Discussion

Based on human studies and previous preclinical studies, it is hypothesized that prolactin plays a role in migraine pathology, primarily in women [4, 5, 8, 9, 16]. The current preclinical study expands the understanding of the potential mechanisms that prolactin may use to contribute to migraine. Here, we demonstrate that both immune cells (specifically macrophages) and iNOS can act downstream of prolactin signaling in response to dural administration of prolactin and separately in response to repeated stress exposure. Our prior work has already demonstrated that pituitary prolactin release and the expression of prolactin receptors on peripheral sensory neurons are important for migraine-like behavioral responses to repeated stress, although only in females [9], and we also observe a female-specific role of iNOS in response to stress here. Together with prior work, these new data suggest that the immune system and macrophages in particular may be important for the effects of prolactin in migraine.

Previous in vitro studies found that prolactin transiently increased capsaicin-evoked responses through PKCϵ or PI3K pathways in female rat TG neurons [46]. Additionally, prolactin selectively sensitized sensory neurons collected from female mice and female human donors [47]. This female-specific response could result from the greater expression of prolactin receptors on sensory nerve endings within the adult female dura in comparison to males [8]. Together, these studies confirm the sensitizing effect of prolactin on female TG neurons. However, it is widely recognized that prolactin receptors are not specifically expressed on neurons but are broadly expressed on many cell types. Our findings here illustrate that the selective deletion of prolactin receptors from Nav1.8-positive sensory neurons fails to completely inhibit dural prolactin-induced periorbital hypersensitivity (Fig. 1A). This suggests that prolactin-evoked responses are mediated by multiple cell types and not only mediated by receptors in the TG Nav1.8-positive afferents. Our prior studies using this same Nav1.8^cre/−^/ Prlr^fl/fl^ mouse line showed a lack of expression of prolactin receptors on Nav1.8-positive neurons [9]. Prior work using Nav1.8 cre mice found that the channel is expressed in 75% of sensory neurons [48] so the prolactin receptor would be deleted from the vast majority of neurons in sensory ganglia. Nonetheless, it is possible that the partial loss of dural prolactin response is due to deletion of receptors only in this population. However, we favor the explanation that other cell types contribute to these responses, especially given the additional supportive evidence shown using macrophage depletion.

Our data support that macrophages are essential for dural prolactin responses since depletion of macrophages using the standard clodronate liposome protocol led to a significant loss of migraine-like behavior following dural injections (Fig. 1B). Due to the systemic administration protocol, macrophages would be depleted throughout the body so one caveat of this finding is that we are not able to conclude that the effects of clodronate liposomes are solely attributable to depletion of dural macrophages. Prolactin is known to be involved in more than 300 separate functions, and its receptors have been found in numerous cell types [49]. Prolactin affects several types of immune responses, such as activation of both the innate and adaptive immune system, stimulating the release of inflammatory cytokines and reactive oxygen species [29–31, 50, 51]. In the dura mater where prolactin was applied, multiple studies utilizing diverse methodologies have consistently revealed, across various species, that macrophages constitute the predominant population of immune cells within dura mater [25, 44, 52]. Application of prolactin to macrophages resulted in increased release of NO, superoxide, nitrogen dioxide, and inflammatory cytokines [26–28, 53, 54]. Prior research supports a role for both NO and reactive oxygen species (ROS) in the underlying mechanisms of migraine [55–57]. Prolactin exposure also increased iNOS expression levels in macrophages, consistent with increased NO release [29]. Here, we demonstrated the contribution of iNOS in the development of dural prolactin-induced periorbital hypersensitivity (Fig. 2). AR-C102222 inhibits iNOS with an IC50 of 35 nM, and has 3,000-fold selectivity over epithelial NOS (eNOS) and 20-fold selectivity over neuronal NOS (nNOS) [58]. However, considering ARC’s rapid effects and lack of full selectivity for iNOS, nNOS may also be blocked by this compound, and thus the periorbital hypersensitivity at early timepoints could be mediated by the neuronal isoform. Additionally, while we cannot conclusively determine that the location of iNOS that is relevant for these mechanisms is within macrophages, the data are consistent with macrophages being a major location for iNOS production, and an increase in iNOS activity following prolactin exposure in prior studies. Future work with targeted deletion of iNOS from macrophages would be needed to more clearly demonstrate a role of macrophage-derived iNOS in the effects of dural prolactin. Nonetheless, these new findings provide supportive evidence of the involvement of immune responses within the meninges in prolactin-induced hypersensitivity.

Another main finding of this work is that macrophages contribute to the migraine-like behavioral responses following repeated stress exposure. Using the same clodronate liposome protocol to deplete macrophages as in the dural prolactin studies, we found that depletion of macrophages either before or after repeated stress exposure attenuated the behavioral responses to stress, including the primed response to SNP (Figs. 3 and 4). Because we have shown previously that repeated stress exposure leads to migraine-like behavioral responses in both males and females, we tested both sexes here, and the effects of macrophage depletion were similar between sexes. This suggests that macrophages play a crucial role in stress-related behaviors regardless of sex. However, given the impact of stress on the entire animal, it is unclear where macrophages are contributing in this behavioral model. Aside from the dura mater, macrophages are found in TG [59, 60], and previous studies showed the crosstalk in TG between macrophages and sensory neurons [27]. In fact, subdural macrophages are also activated by cortical spreading depression, suggesting their involvement in migraine pathophysiology [22]. It is also possible that macrophages outside of the trigeminal afferent system contribute to behavioral responses to stress and NO donors as both stimuli can act throughout the body.

Prior studies found that there were more macrophages in naïve female dura than males. Following chronic stress, macrophage levels significantly increased in males, but not females, reaching a level comparable to that observed in females [45]. This may suggest that while macrophages are important for stress responses in both sexes, their location, population numbers, activation mechanisms, and functions may differ between males and females. We have shown previously that stress significantly increased plasma prolactin levels and prolactin has a female-specific influence on stress-induced behavioral responses [9]. It is possible that prolactin produces its effects, at least in part, through macrophages in females but other factors engage macrophages in males.

While depletion of macrophages before or after stress attenuated behavioral responses in both sexes, surprisingly iNOS inhibition either before or after stress only blocked stress responses in females (Figs. 5 and 6). The explanation for this sex-specific effect is not clear, especially if macrophages are the major source of iNOS that contributes to stress responses. Previous research found that chronic stress significantly increased iNOS RNA levels in myeloid-derived (which include macrophages) immune cells [45]. In females, chronic stress significantly increased T cells in the dura mater but not macrophages [44]. Although the total number of macrophages remained unchanged, increased T cells may upregulate iNOS in macrophages. Notably, Type 1 T cells characteristically produce interferon γ which can strongly activate macrophages to produce high concentrations of nitric oxide via iNOS [61]. Prolactin exposure also activates macrophages leading to increased expression and activity of iNOS [26–28], which may be an additional factor strengthening the role of iNOS in females. While our preclinical data shown here suggest inhibition of iNOS may be effective in migraine, two clinical trails have previously investigated the selective iNOS inhibitor GW274150, both as acute [62] or preventive [63] treatment. These trials failed to demonstrate efficacy compared to placebo. Reasons for these failures are unclear but the effects of these compounds are dependent on nicotinamide adenine dinucleotide phosphate (NADPH) [64] and NADPH levels may change in migraine thus influencing efficacy [65]. Nonetheless, previous human studies highlighted a potential link between increased iNOS activity and migraine [66–68], and when combined with our data, this may suggest a role for iNOS inhibition only in specific migraine sub-populations, such as female patients with migraine with aura [68].

How macrophages contribute to stress responses in males is unclear if there is no role for iNOS. Several clinical studies have found that interleukin 6 (IL-6), a pro-inflammatory cytokine, is elevated in people with migraine compared to healthy volunteers [69]. Animal studies showed that dural stimulation with IL-6 induced periorbital hypersensitivity in both males and females without a sex difference [36, 70]. IL-6 has also been found to be significantly increased after chronic stress in dural myeloid cells in both genders [45]. It is thus possible that IL-6 is more strongly involved in stress-induced responses in males. Future work is needed to better determine what mechanisms are used by macrophages in males in response to stress.

This study has several limitations. First, we did not assess the impact of clodronate on non-macrophage cell types. Clodronate liposomes induce macrophage apoptosis via phagocytosis to effectively deplete macrophage within 24–48 h after injection [71, 72]. It is likely that other cell types that phagocytose substances can also be affected by clodronate liposomes. For example, polymorphonuclear neutrophils can phagocytosize clodronate liposomes as well and it may be that the clodronate effects could be partially mediated by the depletion of neutrophils [73]. This would not alter the conclusion that immune cells contribute to the response to dural prolactin and repeated stress exposure, it would simply change the conclusion as to which cell type (or types) of immune cells contribute. Further studies are needed to address this question. The second limitation is that the iNOS inhibitor is capable of targeting iNOS in many different cell types, not only macrophages. iNOS has been found to be expressed in multiple cell types, such as macrophages, T cells and dendritic cells [74, 75]. While iNOS serves as a marker for M1 macrophages, and macrophages constitute the majority of immune cells in the dura [25], we cannot exclude the potential contribution of iNOS from other cell types. The third limitation is that the stress model, including the macrophage depletion studies and the iNOS inhibitor studies, cannot identify the location of action of the interventions given stress impacts the entire body and the clodronate liposomes and iNOS inhibitor were given systemically. Whether the meninges are a primary site of action of stress, immune cell function, and iNOS activity in the behavioral responses with this model are not clear.

The current study demonstrates the involvement of macrophages and iNOS in preclinical migraine models, including dural stimulation with prolactin and repeated stress. Together with prior studies and the data shown here, we propose that prolactin plays a role in the pain phase of migraine not only by sensitizing TG afferents but also through activation of macrophages and induction of iNOS activity. Importantly, we find that macrophages are required to fully establish stress-induced behavioral responses in both sexes. While the underlying mechanism remains unknown, we show a female-specific role for iNOS activation after repeated stress exposure. Together, these studies further elaborate mechanisms by which prolactin may contribute to migraine in females. Separate studies have indicated that a prolactin antibody is effective against restraint stress-induced hindpaw allodynia and postoperative pain [76, 77], which provides an additional option for targeting prolactin in migraine treatment. Finally, the current studies further support a role for the immune system in both prolactin and stress responses related to migraine and provide a rationale for further investigation of the immune system in this disorder.

## Electronic Supplementary Material

Below is the link to the electronic supplementary material.

**Supplementary Material 1: Supplementary Fig. 1** Depletion of peritoneal macrophages using clodronate liposome injection. (**A**) Mice received two i.p. injections of 150 µl clodronate liposomes (Clod. Liposomes; selective depletion of macrophages) or two i.p. injections of 150 µl of control liposomes (Ctrl. Liposomes) at 48-hours interval. Flow cytometric quantification of peritoneal macrophages 24 h after second injection of clodronate liposomes or control liposomes (n = 2 per group). (**B**) Representative flow cytometry plots of macrophages (CD45⁺F4/80⁺ live cells) in the peritoneal cavity of mice treated with clodronate liposomes. (**C**) Representative flow cytometry plots of macrophages (CD45⁺F4/80⁺ live cells) in the peritoneal cavity of mice treated with control liposomes. * indicates Clodronate Liposomes vs. Ctrl. Liposomes. Significant differences were determined through Unpaired t-tests. Data are represented as mean ± SEM. **<0.01.

## Data Availability

The datasets generated during and/or analyzed during the current study are available from the corresponding author on reasonable request.
