# Supplementary figures and images for: Macrophages and iNOS contribute to the effects of dural prolactin and repeated stress in mouse migraine models
Source: J Headache Pain. 2025 Dec 23;27(1):26. doi: 10.1186/s10194-025-02261-3 (PMC12836902; doi:10.1186/s10194-025-02261-3)

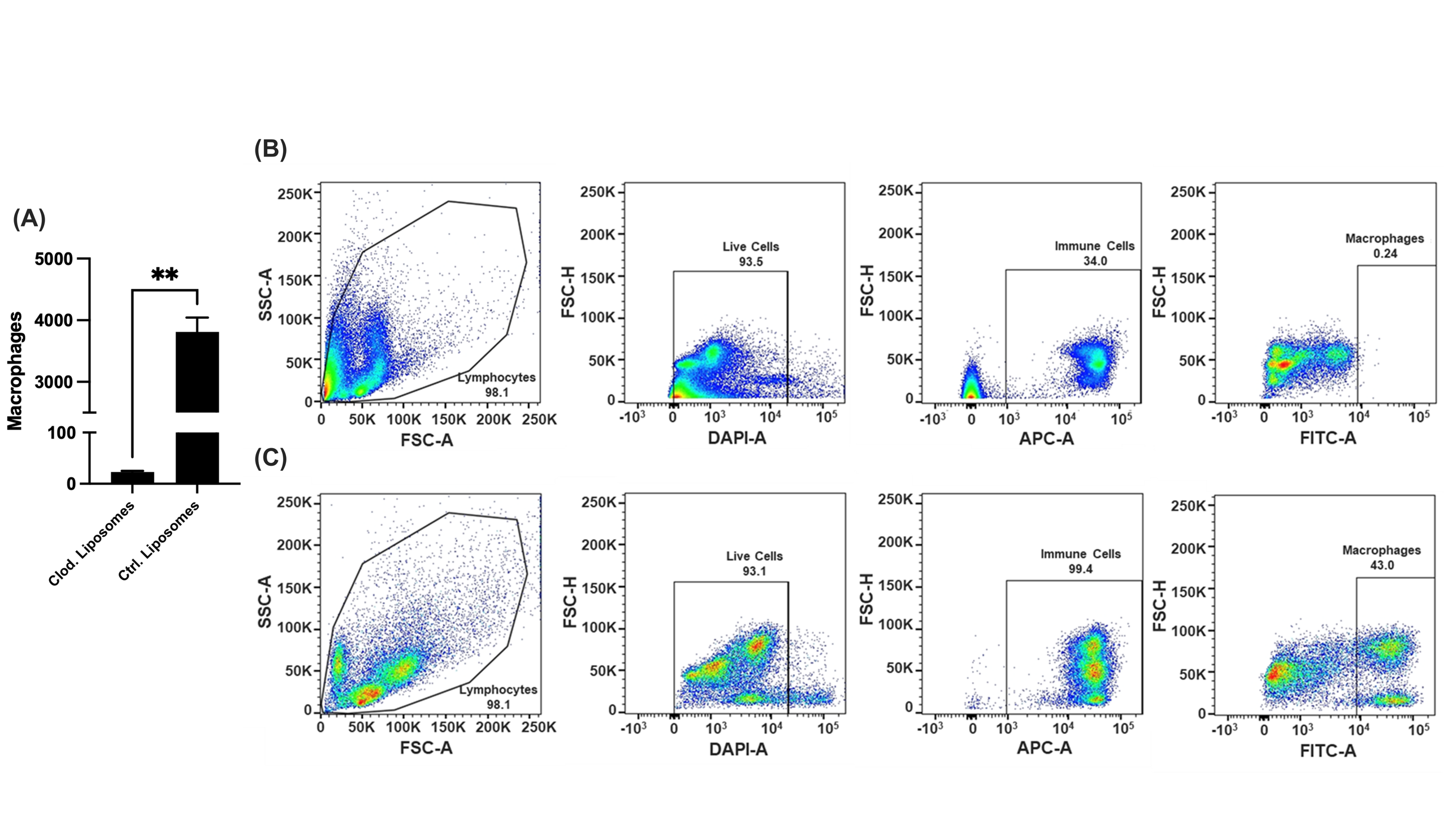

Supplement: Supplementary file 1 — Supplementary Material 1: Supplementary Fig. 1 Depletion of peritoneal macrophages using clodronate liposome injection. (A) Mice received two i.p. injections of 150 µl clodronate liposomes (Clod. Liposomes; selective depletion of macrophages) or two i.p. injections of 150 µl of control liposomes (Ctrl. Liposomes) at 48-hours interval. Flow cytometric quantification of peritoneal macrophages 24 h after second injection of clodronate liposomes or control liposomes (n = 2 per group). (B) Representative flow cytometry plots of macrophages (CD45⁺F4/80⁺ live cells) in the peritoneal cavity of mice treated with clodronate liposomes. (C) Representative flow cytometry plots of macrophages (CD45⁺F4/80⁺ live cells) in the peritoneal cavity of mice treated with control liposomes. * indicates Clodronate Liposomes vs. Ctrl. Liposomes. Significant differences were determined through Unpaired t-tests. Data are represented as mean ± SEM. **<0.01. [file 10194_2025_2261_MOESM1_ESM.tif]
